# The "Massage Establishments" Scandal

**Published:** 1915-10-16

**Authors:** 


### THE "MASSAGE ESTABLISHMENTS" SCANDAL.

### Lords Select Committee's Inquiry and Decision.

A Select Committee of the House of Lords, presided over by the Earl of Kintore, spent two days last week in considering the General Powers Bill promoted by the London County Council. The most important features of the Bill from the public point of view are the clauses which propose to give the County Council powers for the control of lying-in homes and establishments for massage and other forms of treatment, by means of inspection and registration. These clauses were opposed by certain of the borough councils, which contended that, while the principle of inspection and registration was admirable, the work should be entrusted to the borough councils and not to the central authority. Eventually the Committee decided to allow the Bill to proceed in view of the urgency of the circumstances.

Mr. Fitzgerald, K.C., Mr. Clode, K.C., and the Hon. Evan Charteris appeared for the County Council, while the opposing borough councils were represented by Mr. Freeman, K.C., and Mr. Bellewes.

### Lying-in Homes.

Mr. Fitzgerald, in opening the case for the promoters of the Bill, first referred to the necessity of dealing with lying-in homes. He stated that many of those institutions were brought to the notice of unmarried women in various parts of the country who came to London and were nursed in those places. It was only too well known that in some cases women of attractive appearance had been induced to remain in London and to enter upon a life of immorality. The Council did not suggest that there were not lying-in homes which were perfectly properly conducted, but they asked for an extension of their present powers under the Midwives Act so as to include all lying-in homes, whether *bona-fide* or otherwise.

### The Massage Establishments.

Turning to the question of massage establishments, Mr. Fitzgerald said that many places so described in London were a scandal.

Lord Kintore said that no time need be taken up in showing that there were undesirable massage establishments. They were a perfect scandal to-day, and a remedy was urgent. That was the opinion of the whole of the Committee.

Mr. Fitzgerald said that there were a great number of these establishments throughout London. They were extensively advertised by means of sandwichmen who paraded the West End. Some were described as "massage establishments" or "special baths," and others passed under the title of "teachers of languages" or "general deportment." The large majority of them were in reality places where facilities were given for immorality, and into which, under the falsest pretences, young girls were inveigled. No one could question the importance of the matter, especially in view of communications from the Commissioner of Police, which called attention to the powerlessness of the police to cope with it on account of the difficulty of obtaining evidence and the need therefore for official visitation and inspection. The Council asked power, if they had reason to believe that a proper business was not being carried on, to refuse registration, and thus it was hoped the objectionable establishments would be eliminated.

Among those who gave evidence in support of the Bill were Mrs. Wilton Phipps, Chairman of the London County Council Midwives Committee; Dr. Hamer, the Council's Medical Officer; Mr. Percy Simmons, Chairman of the Public Control Committee; Mrs. Gow, and representatives of the Central Midwives Board; the Queen Victoria Jubilee Institute of Nurses; and the Incorporated Society of Trained Nurses. These witnesses strongly urged the desirability of placing the powers in the hands of the County Council rather than of the officials of twenty-eight different borough councils.

### Testimony of Metropolitan Police.

Mr. F. T. Bigham, Assistant Commissioner of the Metropolitan Police, said that the Commissioner strongly felt the desirability of vesting these powers in the County Council. It had been found, on inquiry being made into supposed *bona-fide* massage and bath businesses, that the establishments were carried on by women of doubtful character, usually on the top floors of large buildings. The police had no power, however, to demand inspection, and they encountered the utmost difficulty in obtaining warrants. He knew of a case where a particularly notorious woman was convicted, and, after undergoing imprisonment, continued the business under another name. The police also knew of cases in which young girls of seventeen had been enticed to such places and thenceforward had led immoral lives. While the houses were carried on as they were now it was impossible for the police to get at the root of the evil, but they believed that the evil would be stopped if all establishments advertised as "massage" and "baths" were registered. Mr. Fitzgerald offered to bring evidence from officials of the Council who had visited some of the establishments, but the Committee intimated that they did not desire to hear further evidence, and the case for the promoters of the Bill was closed.

### Grounds of Opposition.

Alderman T. H. R. Evans (Fulham), Chairman of the Metropolitan Boroughs Standing Committee, said that the evil did not come home to the County Council so closely as it did to the local authorities, and he urged that the powers would be better carried out by each borough in its own particular district than by the central body.

Mr. Freeman, K.C., addressing the Committee for the opponents of the measure, contended that the duplication of authorities for the purpose was unnecessary and pernicious and it must throw additional and uncalled for cost upon the ratepayers. The borough councils had better means of tracing those who abused the name of massage establishments than a central body, and they already had the jurisdiction to deal with disorderly houses. The only effect of the Bill, if passed in its present form, would be to create a separate class of disorderly houses while leaving the bulk of them in the hands of the local authorities.

### The County Council or 28 Borough Councils.

Mr. Fitzgerald said that really the only question in dispute was whether the administration of these powers, which everybody admitted to be desirable, should be entrusted to one authority or to twenty-eight. Of the twenty-eight borough councils twelve had not expressed any desire whatever to have the powers, while Islington, one of the largest, did not desire them. He pointed out that those councils which did not want the powers would not be likely to start carrying them out at once in a very active manner. The Bill had been brought forward by the County Council at the suggestion of the Commissioner of Police and the Home Office, while the Incorporated Society of Trained Masseuses supported the Bill on the distinct understanding that the powers sought were to be centred in the County Council.

The Select Committee's Decision.

The Committee then deliberated in private, and when the proceedings were resumed in public the Chairman asked Mr. Fitzgerald whether, in the event of the County Council getting their Bill, they would make use of the services for the purposes of inspection of the officers of the various borough councils. Mr. Fitzgerald replied that he had no instructions on the point, so that he could not answer the question one way or the other.

Lord Kintore then announced that the Committee shared to the full the desire of the County Council and of the borough councils alike that these evils should be promptly suppressed, and considering the urgency of the present circumstances the Committee were of opinion that the Bill should be allowed to proceed. Mr. Freeman suggested that a clause should be inserted similar to that in regard to the notification of births in the Midwives Act, giving power to the County Council to delegate its duties to the borough councils. The Committee agreed to adopt this suggestion, and the proceedings then ended.

